# Functional connectivity along the anterior–posterior axis of hippocampal subfields in the ageing human brain

**DOI:** 10.1002/hipo.23097

**Published:** 2019-05-06

**Authors:** Marshall A. Dalton, Cornelia McCormick, Flavia De Luca, Ian A. Clark, Eleanor A. Maguire

**Affiliations:** ^1^ Wellcome Centre for Human Neuroimaging, UCL Queen Square Institute of Neurology University College London London UK

**Keywords:** ageing, functional connectivity, hippocampal subfields, perirhinal cortex, subiculum, tau

## Abstract

While age‐related volumetric changes in human hippocampal subfields have been reported, little is known about patterns of subfield functional connectivity (FC) in the context of healthy ageing. Here we investigated age‐related changes in patterns of FC down the anterior–posterior axis of each subfield. Using high resolution structural MRI we delineated the dentate gyrus (DG), CA fields (including separating DG from CA3), the subiculum, pre/parasubiculum, and the uncus in healthy young and older adults. We then used high resolution resting state functional MRI to measure FC in each group and to directly compare them. We first examined the FC of each subfield in its entirety, in terms of FC with other subfields and with neighboring cortical regions, namely, entorhinal, perirhinal, posterior parahippocampal, and retrosplenial cortices. Next, we analyzed subfield to subfield FC within different portions along the hippocampal anterior–posterior axis, and FC of each subfield portion with the neighboring cortical regions of interest. In general, the FC of the older adults was similar to that observed in the younger adults. We found that, as in the young group, the older group displayed intrinsic FC between the subfields that aligned with the tri‐synaptic circuit but also extended beyond it, and that FC between the subfields and neighboring cortical areas differed markedly along the anterior–posterior axis of each subfield. We observed only one significant difference between the young and older groups. Compared to the young group, the older participants had significantly reduced FC between the anterior CA1‐subiculum transition region and the transentorhinal cortex, two brain regions known to be disproportionately affected during the early stages of age‐related tau accumulation. Overall, these results contribute to ongoing efforts to characterize human hippocampal subfield connectivity, with implications for understanding hippocampal function and its modulation in the ageing brain.

**Abbreviations**
A: anterior (of the hippocampus)
AB: anterior body (of the hippocampus)
CA 1–4: cornu ammonis 1–4
DG: dentate gyrus
ENT: entorhinal cortex
FC: functional connectivity
PB: posterior body (of the hippocampus)
PHC: posterior parahippocampal cortex
PRC: perirhinal cortex
RSC: retrosplenial cortex
rsfMRI: resting state functional magnetic resonance imaging
T: tail (of the hippocampus)

## 1 INTRODUCTION

Lynn Nadel has had an immense influence on cognitive and memory neuroscience as is clearly evident in this special issue. His work, not only in the realm of spatial representations (O'Keefe & Nadel, 1978), but also autobiographical memory (Ryan et al., 2001), memory consolidation (Nadel & Moscovitch, 1997) and sleep (Payne & Nadel, 2004), has had a wide reach, including being influential on this article's senior author. Indeed, his 1991 article in Hippocampus (Nadel, 1991) appeared at the start of her PhD and was instrumental in directing her to ideas about cognitive maps and to a career seeking an understanding of hippocampal function. Given Nadel's unwavering curiosity coupled with an enviable knowledge of the literature, his prowess as a theoretician and his mentorship that so many of us have enjoyed, his high standing in the field is justly deserved.

Another feature of Nadel's work is its prescience. Many key ideas and concepts which went on to prove important in the field are contained in his classic book with John O'Keefe (O'Keefe & Nadel, 1978). One in particular is the focus of the current study and, in fact, was held by Nadel to be of such relevance for understanding the hippocampus that it was the subject of his PhD—*Behavioral effects of dorsal and ventral hippocampal lesions in the rat* (Nadel, 1967; see also Nadel, 1968). Nadel astutely realised (see also Kimura, 1958; Nauta, 1956) that the dorsal (posterior in humans) and ventral (anterior in humans) hippocampus likely facilitate different functions. At that point he was unable to derive a full explanation for this disparity.

In the five decades since his PhD, many others have gone on to note this anterior–posterior distinction adding further to the picture, including that the dorsal hippocampus in rats is more associated with spatial processing compared to the ventral (Moser & Moser, 1998), that place fields in the dorsal hippocampus of rats are smaller than those in the ventral hippocampus (Kjelstrup et al., 2008), that the posterior hippocampus in London taxi drivers is enlarged while the anterior hippocampus is decreased in volume (Maguire et al., 2000), and that the anterior human hippocampus seems to be heavily involved in constructing scene imagery (Zeidman & Maguire, 2016). Despite these insights, however, we still lack a clear understanding of why there is this anterior–posterior distinction in hippocampal function. This is likely due in no small part to the issue being more complex than merely a categorical difference. This becomes clear when considering hippocampal anatomy.

The primary input to the hippocampus is via the entorhinal cortex (ENT), the source of the canonical tri‐synaptic pathway. The ENT primarily innervates the dentate gyrus (DG) and, from here, intrahippocampal connectivity is generally acknowledged to follow a unidirectional pathway through the CA regions to the subiculum, the primary region of efferent projection from the hippocampus (Aggleton & Christiansen, 2015; Duvernoy, Cattin, & Risold, 2013). While this canonical circuitry is not in question, noncanonical feedback connections from CA3 to DG, and from subiculum to CA1, have been noted in rodents (Sik, Ylinen, Penttonen, & Buzsaki, 1994; Xu, Sun, Holmes, & López, 2016). Anatomical evidence from nonhuman primates has also shown that extra‐hippocampal regions including the ENT, perirhinal (PRC), posterior parahippocampal (PHC), and retrosplenial (RSC) cortices interact directly with specific hippocampal subfields, bypassing the canonical hippocampal pathway (Aggleton, 2012; Agster & Burwell, 2013; Kobayashi & Amaral, 2007; Leonard, Amaral, Squire, & Zola‐Morgan, 1995; Witter & Amaral, 1991). Moreover, tract tracing studies in nonhuman primates have revealed intrasubfield gradients of connectivity along the anterior–posterior axis of the hippocampus (Insausti & Muñoz, 2001). This suggests that different portions of hippocampal subfields may preferentially interact with other brain regions. This resonates with the known gradual genetic, anatomical, and functional differentiations along the long axis of the hippocampus that have also emerged over recent decades (see Fanselow & Dong, 2010; Poppenk, Evensmoen, Moscovitch, & Nadel, 2013; Strange, Witter, Lein, & Moser, 2014 for reviews).

Until recently, in vivo examination of the connectivity between different subfields, and different portions of subfields, in humans has been beyond the scope of direct scrutiny. However, high resolution magnetic resonance imaging (MRI) now makes these investigations tractable. Specifically, we have the spatial resolution to delineate individual subfields (Dalton, Zeidman, Barry, Williams, & Maguire, 2017; Yushkevich et al., 2015) in order to assess their functions and connectivity, although their connectivity has received much less attention, despite likely being of significant importance in driving anterior–posterior hippocampal differences.

One way to examine subfield connectivity is to characterize patterns of functional connectivity (FC) using resting state functional MRI (rsfMRI). While rsfMRI FC often reflects anatomical pathways, its statistical dependencies are not limited to the underlying anatomy (Honey et al., 2009; Honey, Thivierge, & Sporns, 2010). Thus, rsfMRI FC has the additional benefit of reflecting potential functional relationships between brain regions. In a recent study we used high resolution rsfMRI to interrogate FC in healthy young adults (Dalton, McCormick, & Maguire, 2019). We first analyzed the FC of each hippocampal subfield in its entirety, in terms of FC with other subfields and with neighboring regions, namely ENT, PRC, PHC, and RSC. We also analyzed FC for different portions of each hippocampal subfield along its anterior–posterior axis, in terms of FC between different parts of a subfield, FC with other subfield portions, and FC of each subfield portion with the neighboring cortical regions of interest (ROI). We found that intrinsic FC between the subfields aligned generally with the tri‐synaptic circuit but also extended beyond it. Our findings also revealed that patterns of FC between the subfields and neighboring cortical areas differed markedly along the anterior–posterior axis of each hippocampal subfield.

While these patterns were characterized in healthy young adults, it is widely acknowledged that there are changes in hippocampal structure and function during healthy ageing. Given the ageing population of the western world, understanding the course and correlates of hippocampal ageing assumes increasing significance. To date, the majority of studies that have investigated human hippocampal subfields in the context of healthy ageing have utilized structural imaging and volumetric analysis techniques. Taken together, these studies consistently show age‐related volume reductions in the subiculum (Chetelat et al., 2008; La Joie et al., 2010; Wang et al., 2003; Yang, Goh, Chen, & Qiu, 2013; Ziegler et al., 2012) and CA1 (de Flores et al., 2015; Frisoni et al., 2008; Mueller et al., 2007) although volume reductions have also been noted in other subfields (Pereira et al., 2014). This is interesting in light of post mortem examinations that showed the subiculum and CA1 were the first hippocampal subfields to be affected by age‐related processes (Lace et al., 2009) and neuron loss (Simic, Kostovic, Winblad, & Bogdanovic, 1997; West, Coleman, Flood, & Troncoso, 1994). Of particular note is that, while normally associated with forms of dementia such as Alzheimer's disease, tau protein accumulation is commonly observed in examinations of post mortem brain tissue from individuals who were clinically healthy at death (Davis, Schmitt, Wekstein, & Markesbery, 1999; Knopman et al., 2003). These lines of evidence suggest that the subiculum and CA1 may be particularly vulnerable to age‐related changes even in those who are cognitively healthy.

While some studies have used task‐based fMRI to investigate age‐related differences in hippocampal subfield function (Maass et al., 2014; Suthana et al., 2010; Yassa et al., 2010), recent studies have successfully utilized rsfMRI to examine FC. However, most rsfMRI investigations of age‐related changes in hippocampal FC used seed regions that were not specific to hippocampal subfields. Rather, some utilized larger seed regions that incorporated multiple subfields within a single ROI (Das et al., 2013) or smaller seed regions that likely encompassed portions of different subfields, or were unclear as to whether they were restricted or not to a specific subfield (Damoiseaux, Viviano, Yuan, & Raz, 2016). Only a few ageing studies have used hippocampal subfields as seed regions in FC analyses (Bai et al., 2011; de Flores et al., 2017; Wang et al., 2015). In most cases, the focus was on disease‐related changes in hippocampal FC. To the best of our knowledge, no study has systematically investigated differences in FC along the anterior–posterior axis of hippocampal subfields in the context of healthy ageing.

The aim of the current study was to conduct such an investigation. Taking into consideration the results of previous investigations of age effects on subfield volume and hippocampal pathology noted above, we predicted that, compared to a group of healthy young adults, healthy older participants would show reduced patterns of rsfMRI FC involving the subiculum and also CA1.

## 2 MATERIALS AND METHODS

### 2.1 Participants

Fifteen young and fifteen older right handed participants took part in the study (young: 6 females, mean age 23.8 years, *SD* 3.1; older: 6 females, mean age 69.6 years, *SD* 4.3). We defined individuals as “older” in this study if they were aged 65 years or above, given that this is the age at which a person can claim the state pension on retirement in the UK. All gave written informed consent to participate in accordance with the University College London research ethics committee. Note that the young adult participants were a completely separate group to that reported by Dalton et al. (2019). The participants were free from any significant health issues and were not taking any medication. They completed the matrix reasoning subtest of the Wechsler Adult Intelligence Scale (WAIS‐IV; Wechsler, 2008) as a measure of general intellectual ability and the Beck Depression Inventory (BDI‐II; Beck, Steer, & Brown, 1996) in order to screen for depression. Results of independent samples *t*‐tests showed that there were no significant differences between the two participant groups on either measure (matrix reasoning *t*[28] = 1.115, *p* = .274; BDI *t*[28] = .734, *p* = .469). We also conducted analyses to examine whether there were any group differences in grey matter volume in any of our ROIs. Analyses (in mm^3^) adjusted for intracranial volume revealed no statistically significant group differences in the volume of any whole subfield, portion of a subfield along the anterior–posterior axis or extra‐hippocampal cortical ROI. The young and older adults were, therefore, well matched. Two subfield ROIs did, however, come close to reaching significance—anterior CA1 (*t*[28] = 1.948, *p* = .057) and the whole uncus (*t*[28] = 1.809, *p* = .081), with reduced volume in the older participant group. We return to this point in Section 4.

### 2.2 Data acquisition and preprocessing

Structural and functional MRI data were acquired using a 3T Siemens Trio scanner (Siemens, Erlangen, Germany) with a 32‐channel head coil within a partial volume centered on the temporal lobe that included the entire extent of the temporal lobes and our other ROIs.

Structural images were collected using a single‐slab 3D T2‐weighted turbo spin echo sequence with variable flip angles (SPACE; Mugler 3rd. et al., 2000) in combination with parallel imaging, to simultaneously achieve a high image resolution of ∼500 μm, high sampling efficiency and short scan time while maintaining a sufficient signal‐to‐noise ratio (SNR). After excitation of a single axial slab the image was read out with the following parameters: resolution = 0.52 × 0.52 × 0.5 mm^3^, matrix = 384 × 328, partitions = 104, partition thickness = 0.5 mm, partition oversampling = 15.4%, field of view = 200 × 171 mm^2^, TE = 353 ms, TR = 3,200 ms, GRAPPA x 2 in phase‐encoding (PE) direction, bandwidth = 434 Hz/pixel, echo spacing = 4.98 ms, turbo factor in PE direction = 177, echo train duration = 881, averages = 1.9, plane of acquisition = sagittal. For reduction of signal bias due to, for example, spatial variation in coil sensitivity profiles, the images were normalized using a prescan, and a weak intensity filter was applied as implemented by the scanner's manufacturer. Each scan lasted 12 min. To improve the SNR of the anatomical image, three scans were acquired for each participant, coregistered and averaged. Each structural scan was visually inspected for quality. Where scan quality was compromised due to movement artifacts, it was discarded. We considered participants with two high quality structural scans a minimum requirement for inclusion in the study. Additionally, a whole brain 3D FLASH structural scan was acquired with a resolution of 1 × 1 × 1 mm.

Functional data were acquired using a 3D echo planar imaging (EPI) sequence which has been demonstrated to yield improved BOLD sensitivity compared to 2D EPI acquisitions (Lutti, Thomas, Hutton, & Weiskopf, 2013). Image resolution was 1.5 × 1.5 × 1.5 mm^3^ and the field‐of‐view was 192 mm^2^ in‐plane. Forty slices were acquired with 20% oversampling to avoid wrap‐around artifacts due to the imperfect slab excitation profile. The echo time (TE) was 37.30 ms and the volume repetition time (TR) was 3.65 s. Parallel imaging with GRAPPA image reconstruction (Griswold et al., 2002) acceleration factor 2 along the phase‐encoding direction was used to minimize image distortions and yield optimal BOLD sensitivity. The dummy volumes necessary to reach steady state and the GRAPPA reconstruction kernel were acquired prior to the acquisition of the image data as described in Lutti et al. (2013). Correction of the distortions in the EPI images was implemented using B0‐field maps obtained from double‐echo FLASH acquisitions (matrix size 64 × 64; 64 slices; spatial resolution 3 × 3 × 3 mm^3^; short TE = 10 ms; long TE = 12.46 ms; TR = 1,020 ms) and processed using the FieldMap toolbox in SPM (Hutton et al., 2002). Two hundred and five volumes were acquired with the scan lasting just under 13 min.

Preprocessing of structural and rsfMRI data was conducted using SPM12 (http://www.fil.ion.ac.uk/spm). All images were first bias‐corrected, to compensate for image inhomogeneity associated with the 32 channel head coil (van Leemput, Maes, Vandermeulen, & Suetens, 1999). Fieldmaps were collected and used to generate voxel displacement maps. EPIs were then realigned to the first image and unwrapped using the voxel displacement maps calculated above. The two/three high‐resolution structural images were averaged to reduce noise, and co‐registered to the whole brain structural FLASH scan. EPIs were also co‐registered to the whole brain structural scan. In order to keep the EPI signal within each hippocampal subfield mask as pure as possible no spatial smoothing was applied for these analyses.

### 2.3 Segmentation of hippocampal subfields

**Figure 1:**
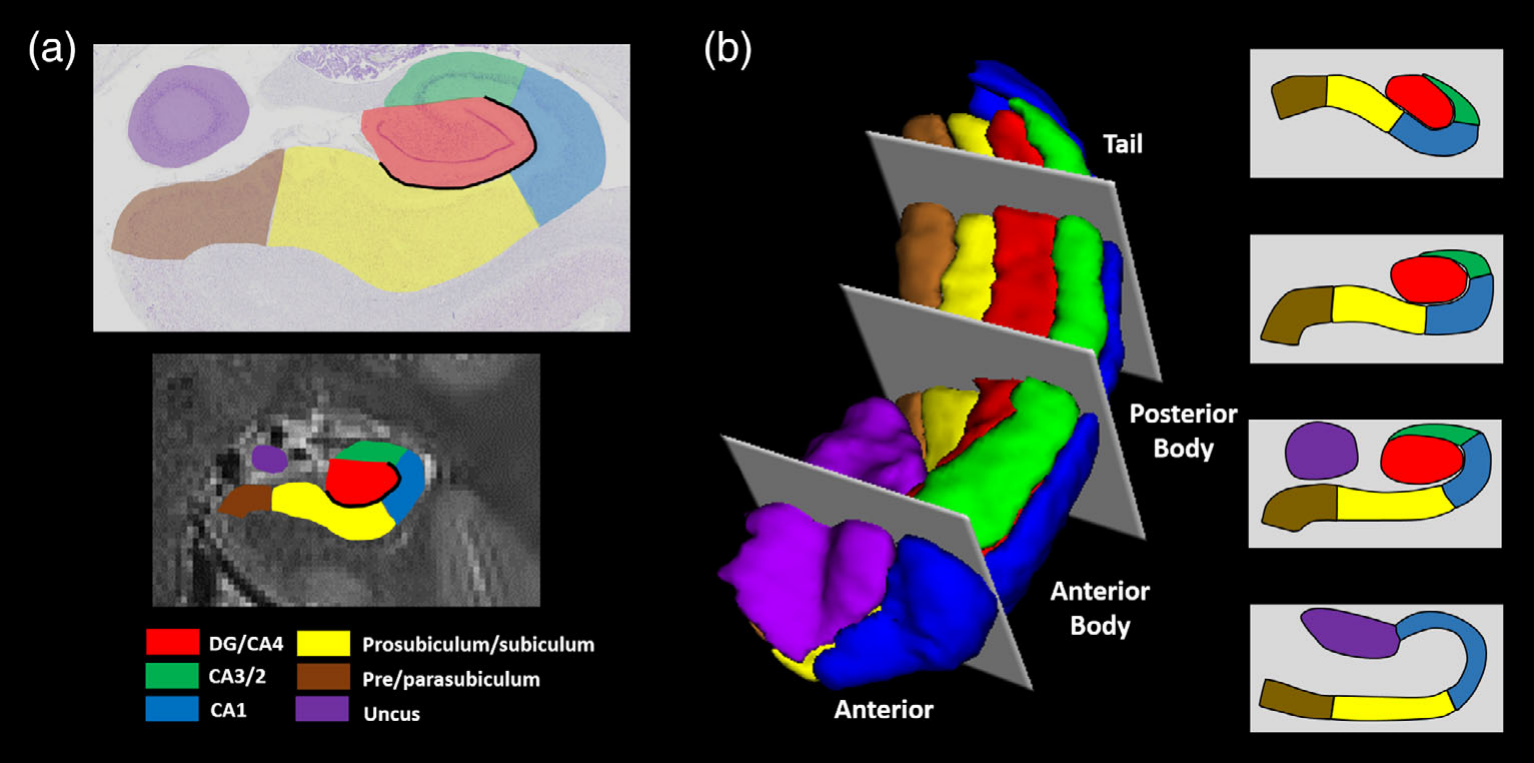
Subregions of the human hippocampus. (a) Top panel: a section of postmortem human hippocampus stained with cresyl violet to visualise cell bodies and overlaid with hippocampal subregion masks. Bottom panel: a T2‐weighted structural MRI scan of the human hippocampus overlaid with hippocampal subregion masks. (b) Left panel: a 3D model of hippocampal subregion masks with representative examples of demarcation points for anterior, anterior body, posterior body and tail portions of the subfields. Right panel: schematic representation of the subfields present in each portion of the hippocampus [Color figure can be viewed at http://wileyonlinelibrary.com]

For each participant, we first manually delineated hippocampal subfields, bilaterally, on native space high resolution structural images according to the methodology described by Dalton et al. (2017) using the ITK Snap software version 3.2.0 (Yushkevich et al., 2006). Masks were created for the following subregions: DG/CA4, CA3/2, CA1, subiculum, pre/parasubiculum, and uncus (Figure 1a). Subfield segmentations were conducted by three researchers (M.A.D., C.M., and F.D.L.). To assess inter‐rater reliability, each researcher independently segmented the hippocampi of the same five participants and analyses for each subfield were conducted using the Dice overlap metric (Dice, 1945) to produce a score between 0 (no overlap) and 1 (perfect overlap). Inter‐rater reliability was 0.84 for DG/CA4, 0.67 for CA3/2, 0.76 for CA1, 0.75 for subiculum, 0.69 for pre/parasubiculum and 0.82 for the uncus. These values are equivalent to those reported in the extant literature (e.g., Bonnici et al., 2012; Palombo et al., 2013). Following this, to allow investigation of FC for different portions of each subfield along the longitudinal axis of the hippocampus, we divided each subfield either into 4 (for CA1, subiculum and pre/parasubiculum), into 3 (for DG/CA4 and CA3/2) or into 2 (for the uncus) separate sections along its longitudinal axis (anterior (A), anterior body (AB), posterior body (PB), and tail (T); Figure 1b) according to the methodology described by Dalton et al. (2019).

To summarize, the often‐used method of using the final slice of the uncus as a demarcation point for anterior and posterior hippocampus (Zeidman, Lutti, et al., 2015; Poppenk et al., 2013), while anatomically useful, may be problematic from a functional perspective. We have consistently observed a functional cluster in the medial hippocampus which extends across this demarcation point in tasks relating to scene‐based cognition (Dalton, Zeidman, McCormick, & Maguire, 2018; Zeidman, Lutti, et al., 2015; Zeidman, Mullally, et al., 2015; Zeidman & Maguire, 2016). Hence, we believe that this portion of the hippocampus may represent a functional module which, when utilizing the uncus‐based anatomical demarcation point, would potentially be split between two separate ROIs. We, therefore, developed a method which allowed us to sample broad portions of each subfield while ensuring this region was kept intact. For the A masks, the anterior boundary was the first slice of the hippocampus and the posterior boundary was the slice preceding the first slice of the DG. This resulted in a mean of 14.4 (*SD* 3.1) slices in the A mask for the older participants and 15.9 (*SD* 3.3) slices for the younger participants. The T mask encompassed the posterior most 15 slices of the hippocampus. We had initially planned to use the crus of the fornix as the anterior demarcation for the T masks but found that, due to individual variability in hippocampal morphology and flexure of the posterior hippocampus, this resulted in some participants having very few slices within the T mask. In order to ensure that the T mask contained an equivalent number of slices across participants we set the anterior most slice of the posterior portion to 15 slices anterior to and including the final slice of the hippocampus. The remaining slices were summed and split in half to create the AB and PB masks. This resulted in a mean of 24.1 (*SD* 3.2) and 23.1 (*SD* 1.9) slices in the AB for older and younger participants respectively, and a mean of 23.7 (*SD* 3.2) and 22.4 (*SD* 2.1) slices in the PB for older and younger participants, respectively. Importantly, results of independent samples t‐tests showed that there were no significant differences between the two participant groups in the number of slices in the A (*t*[28] = 1.295, *p* = .206), AB (*t*[28] = 1.011, *p* = .321), or PB (*t*[28] = 1.324, *p* = .196) portions of the hippocampus. Structural volumes (in mm^3^) for each hippocampal subfield portion for each participant group are provided in Supporting Information Table S1.

### 2.4 Segmentation of extra‐hippocampal ROIs

The ENT, PRC, and PHC were segmented using the guidelines laid out by Augustinack et al. (2013), Fischl et al. (2009) and Berron et al. (2017). The anterior portions of ENT and PRC were generally prone to signal dropout on the fMRI scans. We, therefore, only included posterior portions of these subfields in our analyses. To segment the RSC, we used the cytological investigation of the human RSC by Vogt, Vogt, Perl, and Hof (2001) and the Allen Brain Atlas http://atlas.brain-map.org to gain insights into the likely location of the RSC in the human brain. Of note, this mask only encompassed the thin strip of RSC lying posterior to the corpus callosum and did not include the posterior cingulate cortex, which is commonly conflated with the RSC in neuroimaging investigations. Only ventral portions of the RSC were included owing to the partial volume.

### 2.5 Data analysis

All analyses were performed using the CONN toolbox version 14 for rsfMRI (http://www.nitrc.org/projects/conn). The data were temporally bandpass filtered (0.01–0.1 Hz) and corrected for white matter and ventricular signal. To create FC matrices, time series of voxels within each of the ROIs were averaged and correlated with the averaged time series of all other ROIs resulting in correlation coefficients which were then transformed using Fisher's *z* calculation. Rather than using simple bivariate correlations, we used semi‐partial correlations which allowed us to identify the “unique” contribution of a given source on a target area. Of note, semi‐partial correlations are computed between unmodified and other residualised variables, essentially regressing out or controlling contributions of additional variables, including the activity in all other ROIs in the analysis. Therefore, for each seed analysis in turn, slightly different values were regressed out, resulting in test statistics that vary marginally in their magnitude. That is, the semi‐partial correlations between source Region A and target Region B might be slightly different from the semi‐partial correlation between source Region B and target Region A. The resulting semi‐partial ROI‐to‐ROI correlation matrices from the native space first‐level analyses were further averaged at the second level in order to examine group effects. Importantly, this ROI‐to‐ROI approach allowed us to test hypotheses regarding FC between each ROI and all other ROIs using minimally preprocessed data (i.e., unsmoothed and not normalized). This approach minimized the mixing of BOLD signal between adjacent subfields. For all analyses, ROI‐to‐ROI results were corrected for multiple comparisons and reported when significant at a level of *p* < .05 false discovery rate (FDR) corrected (Chumbley, Worsley, Flandin, & Friston, 2010). The mean number of functional voxels for each hippocampal subfield portion for each participant group is provided in Supporting Information Table S2.

Note that in all cases analyses were based on bilateral masks. We did not investigate laterality differences in the current study as we did not have specific predictions regarding age‐related changes in left/right hippocampal subfield function in this task‐free FC analysis. This would be interesting to examine in the context of future task‐based FC studies.

## 3 RESULTS

### 3.1 Whole subfield rsfMRI analyses

**Figure 2:**
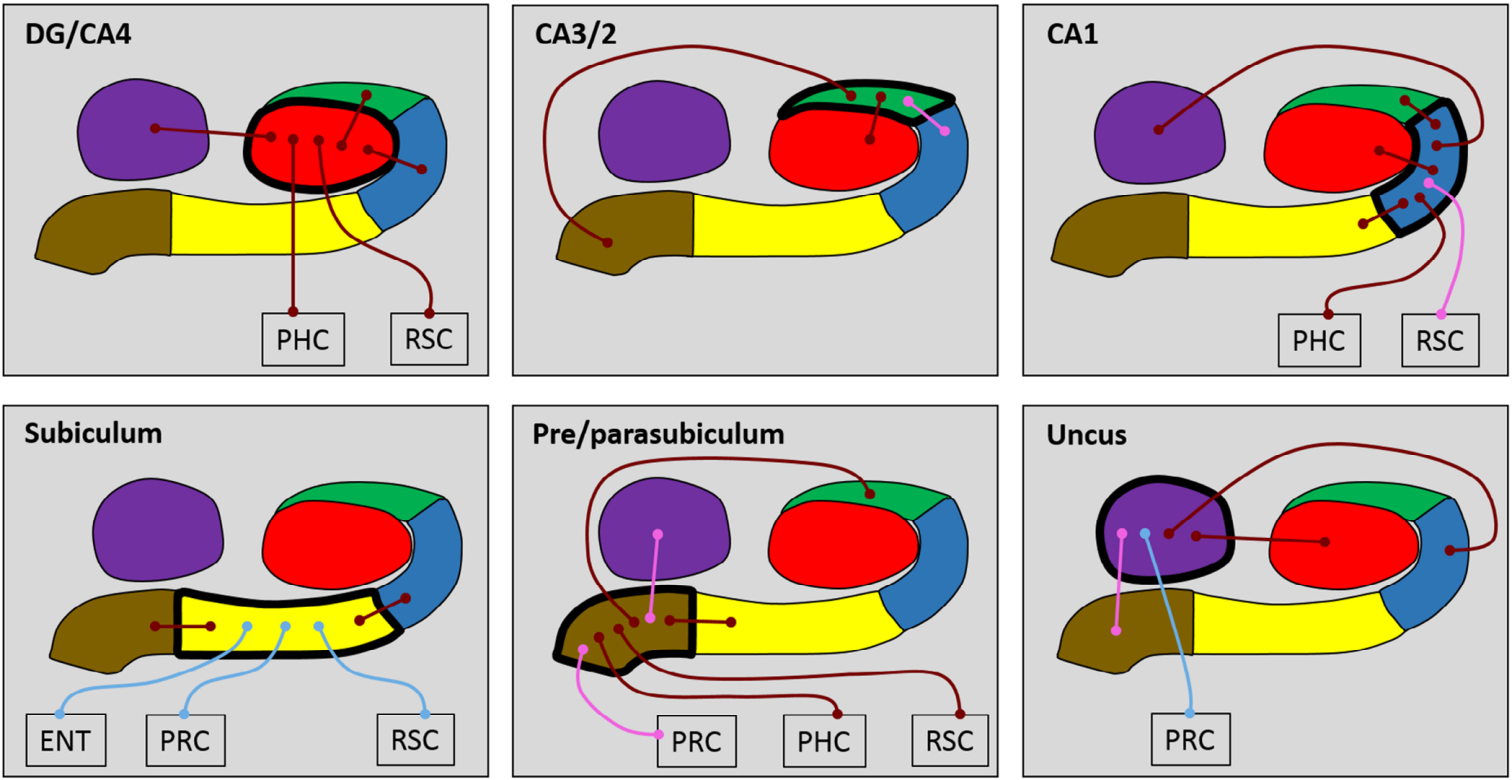
Results of the whole subfield analyses for the young and older participant groups. The relevant subfield in each panel is outlined in a thick black line. The thin lines with circular termini represent significant correlations of activity with the activity in other hippocampal subfields and/or extra‐hippocampal ROIs. Dark red lines represent significant correlations common to both young and old groups. Light blue lines represent significant correlations present only in the young group. Pink lines represent significant correlations present only in the older group. DG/CA4 (red), CA3/2 (green), CA1 (blue), subiculum (yellow), pre/parasubiculum (brown), uncus (purple); ENT, entorhinal cortex; PHC, posterior parahippocampal cortex; PRC, perirhinal cortex; RSC, retrosplenial cortex [Color figure can be viewed at http://wileyonlinelibrary.com]

We first analyzed the FC of each hippocampal subfield in its entirety in terms of FC with other subfields and with the cortical ROIs using 10 bilateral ROIs (DG/CA4, CA3/2, CA1, subiculum, pre/parasubiculum, uncus, ENT, PRC, PHC, and RSC). We initially examined each group (young and older) separately, and then conducted direct between‐group comparisons to investigate age‐related differences in FC. The results of these whole subfield analyses are summarized in Figure 2 and Tables 1 and 2, which also include the statistically significant results of the analyses.

**Table 1:** Statistically significant results of the whole subfield analyses: young participants

Seed ROI	Significant target ROIs	*T*—statistic *t*(28)	*p*—FDR corrected
DG/CA4	CA3/2	9.01	<.0001
	CA1	11.12	<.0001
	Uncus	4.74	<.0001
	Perirhinal cortex	*−4.03*	.0122
	Parahippocampal cortex	3.46	.0306
	Retrosplenial cortex	2.24	.0499
CA3/2	DG/CA4	8.81	<.0001
	Pre/parasubiculum	3.88	.0017
	Uncus	*−5.57*	<.0001
CA1	DG/CA4	10.31	<.0001
	CA3/2	2.24	.0493
	Subiculum	8.42	<.0001
	Pre/parasubiculum	*−2.59*	.0273
	Uncus	3.41	.0045
	Parahippocampal cortex	3.41	.0045
Subiculum	CA1	8.21	<.0001
	Pre/parasubiculum	7.16	<.0001
	Entorhinal cortex	2.54	.0380
	Perirhinal cortex	4.58	.0003
	Retrosplenial cortex	2.33	.0489
Pre/parasubiculum	CA3/2	3.95	.0011
	CA1	*−2.61*	.0257
	Subiculum	7.05	<.0001
	Parahippocampal cortex	7.50	<.0001
	Retrosplenial cortex	4.75	.0002
Uncus	DG/CA4	4.77	.0002
	CA3/2	*−5.65*	<.0001
	CA1	3.25	.0068
	Entorhinal cortex	*−3.71*	.0027
	Perirhinal cortex	2.98	.0106

Negative correlations are shown in italics.

**Table 2:** Statistically significant results of the whole subfield analyses: older participants

Seed ROI	Significant target ROIs	*T*—statistic *t*(28)	*p*—FDR corrected
DG/CA4	CA3/2	9.88	<.0001
	CA1	8.37	<.0001
	Uncus	3.73	.0019
	Perirhinal cortex	*−3.53*	.0026
	Parahippocampal cortex	4.06	.0011
	Retrosplenial cortex	2.36	.0380
CA3/2	DG/CA4	9.95	<.0001
	CA1	3.03	.0117
	Pre/parasubiculum	3.29	.0080
	Uncus	*−4.31*	.0008
CA1	DG/CA4	7.75	<.0001
	CA3/2	2.89	.0109
	Subiculum	8.49	<.0001
	Pre/parasubiculum	*−5.01*	<.0001
	Uncus	6.03	<.0001
	Parahippocampal cortex	4.37	.0003
	Retrosplenial cortex	2.22	.0449
Subiculum	CA1	8.59	<.0001
	Pre/parasubiculum	7.68	<.0001
Pre/parasubiculum	CA3/2	3.42	.0035
	CA1	*−4.96*	<.0001
	Subiculum	7.31	<.0001
	Uncus	2.58	.0232
	Perirhinal cortex	2.31	.0367
	Parahippocampal cortex	5.56	<.0001
	Retrosplenial cortex	4.92	<.0001
Uncus	DG/CA4	3.56	.0040
	CA3/2	*−4.27*	.0009
	CA1	5.49	<.0001
	Pre/parasubiculum	2.47	.0450

Negative correlations are shown in italics.

In young participants, **DG/CA4** was significantly correlated with CA3/2, CA1, uncus, PHC and RSC. This pattern was identical in the older participants.

In young participants, **CA3/2** was correlated with DG/CA4 and the pre/parasubiculum. This pattern was consistent in the older participants with the addition of a correlation with CA1.

In young participants, **CA1** was correlated with DG/CA4, CA3/2, subiculum, uncus, and PHC. This pattern was consistent in the older participants with the addition of a correlation with RSC.

In young participants, **subiculum** was correlated with CA1, pre/parasubiculum, ENT, PRC, and RSC. While intrahippocampal correlations were consistent in the older participants, correlations with extra‐hippocampal ROI's were markedly different to those observed in young participants with no correlation between subiculum and ENT, PRC or RSC in the older group.

In young participants, **pre/parasubiculum** was correlated with the CA3/2, subiculum, PHC, and RSC. This pattern was consistent in the older participants with the addition of a correlation with the uncus and PRC.

In young participants, the **uncus** was correlated with DG/CA4, CA1, and PRC. This pattern was consistent in the older participants with the exception of the correlation with PRC and the addition of a correlation with pre/parasubiculum.

Direct between‐group analyses revealed no significant differences in patterns of FC between young and older participants for any whole subfield or cortical ROI.

These whole subfield results suggest that each hippocampal subfield had a unique pattern of FC with other hippocampal subfields and cortical ROIs. These patterns largely align with our previous report in a separate group of young adult participants (Dalton et al., 2019). Notably, patterns of FC did not differ significantly between the young and older participant groups, although there was a suggestion of less FC between the subiculum and the cortical ROIs in the older participants, which we explored next with more fine‐grained analyses.

### 3.2 Longitudinal axis rsfMRI analyses

**Figure 3:**
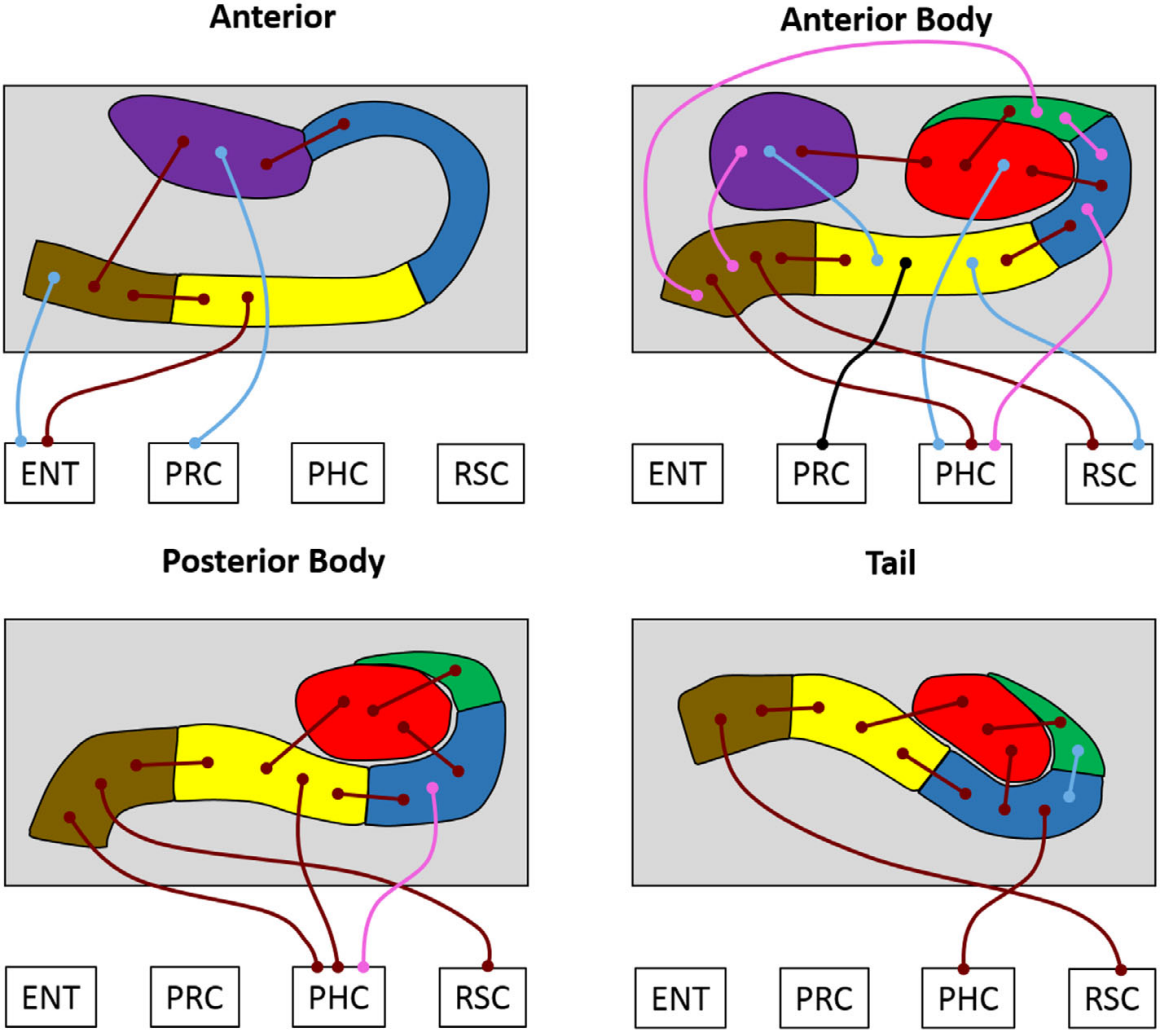
Results of the longitudinal subfield analyses for the young and older participant groups. The thin lines with circular termini represent significant correlations of activity with the activity in other hippocampal subfields and/or extra‐hippocampal ROIs. Dark red lines represent significant correlations common to both young and old groups. Light blue lines represent significant correlations present only in the young group. Pink lines represent significant correlations present only in the older group. The black line represents a significant increase in FC for young compared to older participants. DG/CA4 (red), CA3/2 (green), CA1 (blue), subiculum (yellow), pre/parasubiculum (brown), uncus (purple); ENT, entorhinal cortex; PHC, posterior parahippocampal cortex; PRC, perirhinal cortex; RSC, retrosplenial cortex [Color figure can be viewed at http://wileyonlinelibrary.com]

We next analyzed subfield to subfield FC within different portions of the hippocampus along its anterior–posterior axis, and FC of each subfield portion with the cortical ROIs. We examined this first in the young and older participant groups separately, and then conducted direct between‐group comparisons to investigate age‐related differences in FC. To do this, we performed separate analyses for each portion of the hippocampus: A (8 bilateral ROIs; A CA1, A subiculum, A pre/parasubiculum, A uncus, ENT, PRC, PHC, RSC), AB (10 bilateral ROI's; AB DG/CA4, AB CA3/2, AB CA1, AB subiculum, AB pre/parasubiculum, AB uncus, ENT, PRC, PHC, RSC), PB (9 bilateral ROIs; PB DG/CA4, PB CA3/2, PB CA1, PB subiculum, PB pre/parasubiculum, ENT, PRC, PHC, RSC) and T (9 bilateral ROIs: T DG/CA4, T CA3/2, T CA1, T subiculum, T pre/parasubiculum, ENT, PRC, PHC, RSC). The results are summarized in Figure 3 and Tables 3 and 4, which also includes the statistically significant results of the analyses.

**Table 3:** Statistically significant results of the longitudinal axis subfield analyses: young participants

Seed ROI	Significant target ROIs	*T*—statistic *t*(28)	*p*—FDR corrected
Anterior			
CA1	Uncus	4.54	.0007
	Pre/parasubiculum	*−3.42*	.0068
Subiculum	Pre/parasubiculum	7.36	<.0001
	Entorhinal cortex	3.33	.0085
Pre/parasubiculum	CA1	*−3.45*	.0031
	Subiculum	7.05	<.0001
	Uncus	3.92	.0018
	Entorhinal cortex	3.60	.0029
Uncus	CA1	4.77	.0004
	Pre/parasubiculum	4.17	.0009
	Entorhinal cortex	*−3.92*	.0012
	Perirhinal cortex	3.59	.0022
Anterior body			
DG/CA4	CA3/2	5.26	<.0001
	CA1	13.49	<.0001
	Uncus	8.38	<.0001
	Parahippocampal cortex	3.98	.0010
CA3/2	DG/CA4	4.62	.0007
CA1	DG/CA4	10.94	<.0001
	Subiculum	4.44	.0006
Subiculum	CA1	4.25	.0006
	Pre/parasubiculum	5.69	<.0001
	Uncus	3.73	.0019
	Perirhinal cortex	5.49	<.0001
	Retrosplenial cortex	3.56	.0024
Pre/parasubiculum	Subiculum	5.46	<.0001
	Parahippocampal cortex	7.78	<.0001
	Retrosplenial cortex	3.92	.0016
Uncus	DG/CA4	8.31	<.0001
	Subiculum	3.65	.0048
Posterior body			
DG/CA4	CA3/2	8.69	<.0001
	CA1	8.85	<.0001
	Subiculum	6.84	<.0001
CA3/2	DG/CA4	8.42	<.0001
CA1	DG/CA4	9.23	<.0001
	Subiculum	10.21	<.0001
	Pre/parasubiculum	*−5.73*	<.0001
Subiculum	DG/CA4	6.63	<.0001
	CA1	10.23	<.0001
	Pre/parasubiculum	12.64	<.0001
	Parahippocampal cortex	4.19	.0005
Pre/parasubiculum	CA1	*−5.93*	<.0001
	Subiculum	12.18	<.0001
	Parahippocampal cortex	4.42	.0004
	Retrosplenial cortex	3.65	.0022
Tail			
DG/CA4	CA3/2	9.97	<.0001
	CA1	8.71	<.0001
	Subiculum	4.53	.0003
CA3/2	DG/CA4	9.58	<.0001
	CA1	3.90	.0022
CA1	DG/CA4	9.31	<.0001
	CA3/2	4.22	.0005
	Subiculum	7.75	<.0001
	Parahippocampal cortex	4.70	<.0001
Subiculum	DG/CA4	4.58	.0002
	CA1	8.14	<.0001
	Pre/parasubiculum	7.19	<.0001
Pre/parasubiculum	Subiculum	7.33	<.0001
	Retrosplenial cortex	4.91	<.0001

Negative correlations are shown in italics.

**Table 4:** Statistically significant results of the longitudinal axis subfield analyses: older participants

Seed ROI	Significant target ROIs	*T*—statistic *t*(28)	*p*—FDR corrected
Anterior			
CA1	Uncus	7.46	<.0001
Subiculum	Pre/parasubiculum	6.08	<.0001
	Entorhinal cortex	3.50	.0056
Pre/parasubiculum	Subiculum	5.57	<.0001
	Uncus	4.47	.0004
Uncus	CA1	7.55	<.0001
	Pre/parasubiculum	4.78	.0002
Anterior body			
DG/CA4	CA3/2	5.56	<.0001
	CA1	10.61	<.0001
	Uncus	7.60	<.0001
CA3/2	DG/CA4	5.38	<.0001
	CA1	3.70	.0028
	Pre/parasubiculum	3.72	.0028
CA1	DG/CA4	10.08	<.0001
	CA3/2	3.68	.0018
	Subiculum	3.78	.0018
	Pre/parasubiculum	*−3.70*	.0018
	Parahippocampal cortex	5.99	<.0001
Subiculum	CA1	3.41	.0090
	Pre/parasubiculum	6.52	<.0001
Pre/parasubiculum	CA3/2	3.80	.0016
	CA1	*−3.12*	.0062
	Subiculum	6.92	<.0001
	Uncus	4.16	.0008
	Parahippocampal cortex	3.55	.0025
	Retrosplenial cortex	4.34	.0008
Uncus	DG/CA4	8.31	<.0001
Posterior body			
DG/CA4	CA3/2	8.65	<.0001
	CA1	8.36	<.0001
	Subiculum	6.19	<.0001
CA3/2	DG/CA4	8.50	<.0001
CA1	DG/CA4	8.82	<.0001
	Subiculum	9.87	<.0001
	Pre/parasubiculum	*−4.95*	<.0001
	Parahippocampal cortex	4.53	.0002
Subiculum	DG/CA4	6.34	<.0001
	CA1	9.68	<.0001
	Pre/parasubiculum	11.37	<.0001
	Parahippocampal cortex	4.24	.0004
Pre/parasubiculum	CA1	*−5.08*	<.0001
	Subiculum	11.66	<.0001
	Parahippocampal cortex	3.60	.0024
	Retrosplenial cortex	4.26	.0006
Tail			
DG/CA4	CA3/2	12.41	<.0001
	CA1	8.92	<.0001
	Subiculum	3.40	.0054
CA3/2	DG/CA4	12.15	<.0001
CA1	DG/CA4	9.31	<.0001
	Subiculum	7.79	<.0001
	Parahippocampal cortex	4.71	.0002
Subiculum	DG/CA4	3.28	<.0001
	CA1	8.28	.0074
	Pre/parasubiculum	8.57	<.0001
Pre/parasubiculum	Subiculum	9.25	<.0001
	Retrosplenial cortex	3.72	.0035

Negative correlations are shown in italics.

#### 3.2.1 Anterior

In young participants, activity in CA1 was significantly correlated with the uncus. Subiculum was correlated with pre/parasubiculum and ENT. Pre/parasubiculum was correlated with subiculum, uncus and ENT. The uncus was correlated with CA1, pre/parasubiculum and PRC. These patterns were consistent with those in the older participants, with the exception of the correlations between pre/parasubiculum‐ENT and uncus‐PRC, which were not significant in the older participants. No additional correlations were observed in the older group.

No statistically significant between‐group differences were observed.

#### 3.2.2 Anterior body

In young participants, activity in DG/CA4 was significantly correlated with CA3/2, CA1, uncus, and PHC. CA3/2 was correlated with DG/CA4. CA1 was correlated with DG/CA4 and subiculum. Subiculum was correlated with CA1, pre/parasubiculum, uncus, PRC, and RSC. Pre/parasubiculum was correlated with subiculum, PHC, and RSC. The uncus was correlated with DG/CA4 and subiculum. These patterns were consistent in the older participants with the exception of the correlations between DG/CA4‐PHC, subiculum‐uncus, subiculum‐PRC, subiculum‐RSC which did not reach significance in the older participants. By contrast, significant correlations between CA3/2‐CA1, CA3/2‐pre/parasubiculum, CA1‐PHC, and pre/parasubiculum‐uncus were evident which were not observed in the younger group.

**Figure 4:**
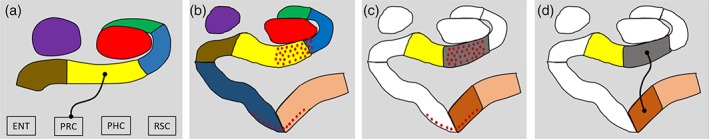
Exploratory analysis. (a) Results of the contrast of the young > older group for the AB hippocampus revealing the subiculum had reduced FC with the PRC in the older participants (thin black line with circular termini). DG/CA4 (red), CA3/2 (green), CA1 (blue), subiculum (yellow), pre/parasubiculum (brown), uncus (purple); ENT, entorhinal cortex; PHC, posterior parahippocampal cortex; PRC, perirhinal cortex; RSC, retrosplenial cortex. (b) Representation of our original segmentation scheme overlaid with red dots representing areas implicated in early (Stage 1) tau accumulation (adapted from Lace et al., 2009). Note the pattern of tau accumulation is largely restricted to the CA1‐subiculum transition region (predominantly within our subiculum mask) and the transentorhinal cortex (predominantly within our perirhinal cortex mask) during these early stages. (c) Representation of our amended segmentation scheme to create ROIs for the putatively tau‐affected CA1‐subiculum transition zone (grey) and transentorhinal cortex (rust). Amended ROIs for the medial subiculum (yellow) and lateral perirhinal cortex (coral) are also displayed. (d) Results for the contrast of the young > older group revealed the CA1‐subiculum transition region had reduced FC with the transentorhinal cortex in the older participants (thin black line with circular termini) [Color figure can be viewed at http://wileyonlinelibrary.com]

There was one significant between‐groups difference—compared to the young participants, older participants had significantly less FC between the subiculum and PRC (*t*[28] = 3.02, *p* = .048 FDR corrected; Figure 3 and Figure 4a).

#### 3.2.3 Posterior body

In young participants, activity in DG/CA4 was significantly correlated with CA3/2, CA1, and subiculum. Activity in CA3/2 was correlated with DG/CA4. CA1 was correlated with DG/CA4 and subiculum. Subiculum was correlated with DG/CA4, CA1, pre/parasubiculum and PHC. Pre/parasubiculum was correlated with subiculum, PHC, and RSC. These patterns were consistent in the older participants, with one additional correlation observed in this group between CA1 and PHC.

No statistically significant between‐group differences were observed.

#### 3.2.4 Tail

In young participants, activity in DG/CA4 was significantly correlated with CA3/2, CA1, and subiculum. CA3/2 was correlated with DG/CA4 and CA1. CA1 was correlated with DG/CA4, CA3/2, subiculum, and PHC. Subiculum was correlated with DG/CA4, CA1, and pre/parasubiculum. Pre/parasubiculum was correlated with subiculum and RSC. These patterns were consistent in the older participants with the exception of the correlation between CA3/2‐CA1 which did not reach significance in this group.

No statistically significant between‐group differences were observed.

Overall, these patterns largely align with those reported in our recent investigation of FC along the anterior–posterior axis of hippocampal subfields in a separate group of young adult participants (Dalton et al., 2019). Our results support the idea that different portions of hippocampal subfields along the anterior–posterior axis of the hippocampus have unique patterns of connectivity with other subfields and extra‐hippocampal cortical ROIs. One difference emerged when the young and older groups were directly compared in the AB portion of the subiculum. Specifically, compared to the young group, the older group showed weaker FC between the AB subiculum and PRC.

Of note, there are numerous ways in which these data could be analyzed. Here we focused our analyses within each portion of the hippocampus, as this was the most efficient way to consider the data and the direct between‐group comparisons. We also conducted additional analyses to investigate differences in FC along the longitudinal axis of each subfield between the young and the older subjects. For each subfield, we included the anterior–posterior portions of that subfield (i.e., A, AB, PB, and T) and ENT, PRC, PHC, and RSC. As with the results reported above, the only significant between‐group difference was for the AB subiculum and PRC (*t*[28] = 3.02, *p* = .041 FDR corrected).

### 3.3 Further exploratory analysis

This observation of decreased FC between the AB subiculum and PRC in the older group is interesting in light of investigations of brain changes in healthy ageing. Most individuals over the age of 65 express tau pathology in the medial temporal lobes, and the earliest affected regions of tau accumulation during normal ageing are a region encompassing the CA1‐subiculum border and the transentorhinal cortex (TEC) (Lace et al., 2009). In the current study, the CA1‐subiculum border region and the TEC were incorporated predominantly in our subiculum and PRC ROIs, respectively. Taking this into consideration, we wondered whether our observation of decreased FC between the AB subiculum and PRC in older participants may be more strongly associated with the CA1‐subiculum border area and TEC, putatively as a consequence of normal age‐related tau accumulation.

To test this, and guided by the report of Lace et al. (2009), we created four new ROIs encompassing the CA1‐subiculum border (the cortical strip comprising CA1 and the subiculum that lies ventral to the DG/CA4), the adjacent medial portion of the subiculum, and we split the PRC mask into a medial TEC portion and a lateral PRC portion (see Figure 4b,c). We ran additional exploratory analyses within these ROIs. This allowed us to probe whether decreased FC between the AB subiculum and PRC was more specifically associated with any of these subregions. Considering the rationale outlined above, we predicted that the older group would show less FC than the younger participants, specifically between the CA1‐subiculum border and TEC. The only significant between‐group difference was, as predicted, less FC between the CA1‐subiculum border region and TEC in the older participants (*t*(28) = 2.89; *p* = .022 FDR corrected; Figure 4d). FC between the medial subiculum and lateral PRC was not significantly different between the groups (*t*(28) = 0.42, *p* = .74).

## 4 DISCUSSION

Understanding subfield connectivity down the long axis of the human hippocampus may be central to helping address the long‐standing question, highlighted by Nadel and others (Kimura, 1958; Nadel, 1968; Nauta, 1956) more than 50 years ago, as to why the anterior and posterior hippocampus seem to perform different functions. Having demonstrated our ability to study subfield rsfMRI FC previously in healthy young adults (Dalton et al., 2019), here we extended this work by examining the effects of healthy ageing. Specifically, we found no between‐group differences in patterns of FC between young and older participants when considering each subfield in its entirely. However, when a more fine‐grained approach was deployed that involved separately examining the A, AB, PB, and T portions of each hippocampal subfield, a group difference emerged. We observed age‐related reductions in FC specifically in the AB portion of the hippocampus, where the older group had reduced FC between the AB subiculum and PRC compared to the younger participants. Additional exploratory analyses revealed that reduced FC between the AB subiculum and PRC may be predominantly associated with decreased FC between the CA1‐subiculum transition region and the TEC, two brain regions known to be disproportionately affected during the early stages of age‐related tau accumulation.

Considering first how the current findings relate to those from our previous investigation of rsfMRI FC in hippocampal subfields in healthy young adults (Dalton et al., 2019), the two sets of results were similar. In this new group of young adults we found, as did Dalton et al. (2019), that intrinsic FC between the subfields aligned generally with the tri‐synaptic circuit but also extended beyond it. Patterns of FC between the subfields and neighboring cortical areas differed markedly along the anterior–posterior axis of each hippocampal subfield. The consistency of findings across two studies shows these effects are replicable and robust.

It is also notable that for both the whole subfield and longitudinal axis analyses, patterns of hippocampal subfield FC in the older participant group generally mirrored the patterns observed in the young participants. This suggests that the dynamics of hippocampal subfield rsfMRI FC may not differ greatly in the context of healthy ageing. This is perhaps not surprising given that our young and older groups were well‐matched on a range of factors that could have affected the FC findings. For example, all participants were healthy and medication‐free, of similar intellectual ability and, while perhaps surprising, there were no volume differences between the groups for any of the ROIs, hence FC differences could not be attributed to partial volume effects. In a sense, this study with its high functioning healthy agers is perhaps the best case scenario in terms of finding minimal effects of age on subfield FC. Nevertheless, even within this context, a significant reduction in AB subiculum connectivity with PRC was apparent.

While the specific functions of the subiculum remain a matter of debate, it is well characterized as the primary output structure of the hippocampus (Duvernoy et al., 2013). Some suggest it may be the heart of the extended hippocampal system (Aggleton & Christiansen, 2015). Our observation of reduced subicular FC in the older participant group aligns with a general consensus that the subiculum may be specifically prone to healthy age‐related changes. Post mortem investigations show that the subiculum and CA1 regions suffer a linear loss of neuron numbers as a function of ageing (Simic et al., 1997; West et al., 1994), and volumetric analyses of structural MRI scans have consistently confirmed age‐related volume reductions in the subiculum and CA1 (Chetelat et al., 2008; de Flores et al., 2015; Frisoni et al., 2008; La Joie et al., 2010; Mueller et al., 2007; Wang et al., 2003; Yang et al., 2013; Ziegler et al., 2012). The subiculum, therefore, appears to be particularly sensitive to the effects of ageing.

It was surprising, therefore, that we did not observe statistically significant between‐group differences in CA1 or subiculum volume in the present study. While not reaching significance, the A CA1 and whole uncus ROIs did show a trend for volume reduction in the older participant group. Our novel method of separating the uncus from the typical hippocampus may offer an explanation for why the expected patterns of age‐related atrophy to CA1 and the subiculum did not reach significance. Extant hippocampal segmentation schemes generally extend hippocampal subfield ROIs into the uncus to include both ‘typical’ and “uncal” portions of a subfield (see Adler et al., 2014; Iglesias et al., 2015; Wisse et al., 2012). In contrast, and in line with Dalton et al. (2017), we created a separate ROI for the uncus, thereby splitting the “uncal” and “typical” portions of CA1 and subiculum between different ROIs. We believe this is a better reflection of the underlying cytoarchitecture. As more researchers adopt this segmentation protocol, it will be interesting to see if, and how, this affects reports of volume differences in ageing. Of note, our goal here was to investigate functional rather than structural differences. Grey matter volume is not always a good proxy for function, given that there are patient cases where volume is reduced yet function is preserved (e.g., Maguire, Kumaran, Hassabis, & Kopelman, 2010), and vice‐versa. Volume and function, therefore, are not necessarily in a linear relationship.

In addition to cell loss and volume reduction, the subiculum is affected by another age‐related process. In the context of the current study, this provides a potential explanatory mechanism for our observation of an age‐related reduction of FC specifically between the AB subiculum and PRC. While commonly linked with Alzheimer's disease, tau protein accumulation also occurs in normal ageing. The slow accumulation of the tau protein results in progressive cell death and subsequent degradation of neuronal communication between affected brain regions. Within the medial temporal lobe, tau accumulation begins in the TEC and spreads, potentially through direct anatomical connections, to the CA1‐subiculum transition area (Lace et al., 2009). These two regions, therefore, are affected during the earliest stages of age‐related tau accumulation. The age‐related reduction in synchronicity between the CA1‐subiculum transition area and the TEC that we have observed here dovetails with this known progression of tau pathology (Lace et al., 2009) and another recent report showing that the subiculum was the only subfield to show reduced FC in patients diagnosed with mild cognitive impairment (de Flores et al., 2017). However, whether the weakening of FC between the AB CA1‐subiculum transition area and TEC is definitively a result of age‐related tau in these regions remains speculative and should be probed further in future investigations.

Our findings also highlight another issue that has relevance for future studies. Researchers using spherical seed based techniques to investigate putative functional differences down the hippocampal long axis should ensure that seeds are placed within the same subfield in the anterior and posterior hippocampus. Moreover, in the light of growing evidence, including that presented by us previously (Dalton et al., 2019; see also Plachti et al., 2019) and in the current study, that different regions of hippocampal subfields may have different functional connections, seed‐based methods should endeavor to specify which subfields are encompassed within the seed regions and discuss the results in the context of these subfields. On a related note, the current findings suggest that, in some contexts, it may be advantageous to eschew classical concepts of hippocampal subfields. Given that the CA1‐subiculum transition area appears to be a “hotspot” of anatomical connectivity across mammalian species (Insausti & Muñoz, 2001; Kondo, Saleem, & Price, 2005; Vogt & Pandya, 1987) and is implicated in the early spread of tau pathology before other regions of the hippocampus (Lace et al., 2009), it may be beneficial to investigate this region as a distinct entity.

In conclusion, while we investigated FC of broad portions of each subfield, we do not suggest that FC is segregated in such a coarse manner. Rather, the gradient nature of connectivity along hippocampal subfields is well documented (reviewed in Strange et al., 2014; Poppenk et al., 2013). Our rationale here was that, in line with this gradient, different portions of each subfield would have a greater proportion of neurons functionally interacting with, for example, the cortical ROIs, and this would be reflected in a stronger correlation between their rsfMRI activity. Overall, we suggest that investigating portions of hippocampal subfields may help to achieve a greater understanding of functional differentiation down the long axis of the hippocampus. In addition, this type of approach could potentially be leveraged to identify biomarkers that might facilitate early diagnosis of hippocampal dysfunction inherent to a range of neurodegenerative disorders. In the fifty years since Lynn Nadel first started contemplating the differences between the dorsal and ventral hippocampus, the huge complexity of this issue has become increasingly apparent. Nevertheless, the hope is that with ever‐more sophisticated techniques for examining the brains of humans and nonhumans, the hippocampus will eventually yield its secrets.

## CONFLICT OF INTEREST

The authors declare no conflict of interest.

5

## Supporting information


**Table S1** Structural MRI volumes (mm^**3**^).
**Table S2.** Functional MRI voxels.

## Data Availability

Requests for the data can be sent to e.maguire@ucl.ac.uk.
