# Supplementary material for: Functional connectivity along the anterior–posterior axis of hippocampal subfields in the ageing human brain
Source: Hippocampus. 2019 May 6;29(11):1049–62. doi: 10.1002/hipo.23097 (PMC6849752; doi:10.1002/hipo.23097)
Supplement: Supplementary file 1 — Table S1 Structural MRI volumes (mm3). Table S2. Functional MRI voxels. [file HIPO-29-1049-s001.docx]

**Functional connectivity along the anterior-posterior axis of hippocampal subfields in the ageing human brain**

Marshall A. Dalton, Cornelia McCormick, Flavia De Luca, Ian A. Clark, Eleanor A. Maguire

**Supporting Information**

**Table S1.** Structural MRI volumes (mm**^3^**).

| **Subfield** | **Subfield portion** | **Young mean volume** | **Young SE** | **Older mean volume** | **Older SE** |
| --- | --- | --- | --- | --- | --- |
| **DG/CA4** | Anterior body | 493 | 21 | 468 | 31 |
|  | Posterior body | 368 | 14 | 375 | 27 |
|  | Tail | 159 | 9 | 173 | 14 |
| **CA3/2** | Anterior body | 135 | 6 | 134 | 14 |
|  | Posterior body | 90 | 3 | 94 | 10 |
|  | Tail | 53 | 4 | 55 | 4 |
| **CA1** | Anterior | 235 | 16 | 194 | 12 |
|  | Anterior body | 272 | 10 | 246 | 17 |
|  | Posterior body | 269 | 11 | 274 | 18 |
|  | Tail | 306 | 14 | 286 | 13 |
| **Subiculum** | Anterior | 190 | 11 | 166 | 10 |
|  | Anterior body | 363 | 14 | 373 | 25 |
|  | Posterior body | 321 | 11 | 341 | 14 |
|  | Tail | 132 | 12 | 128 | 8 |
| **Pre/parasubiculum** | Anterior | 90 | 8 | 83 | 7 |
|  | Anterior body | 213 | 8 | 218 | 16 |
|  | Posterior body | 182 | 6 | 193 | 15 |
|  | Tail | 70 | 4 | 70 | 5 |
| **Uncus** | Anterior | 435 | 33 | 364 | 32 |
|  | Anterior body | 450 | 56 | 376 | 42 |

SE = standard error of the mean

**Table S2.** Functional MRI voxels.

| **Subfield** | **Subfield portion** | **Young mean number of voxels** | **Young SE** | **Older mean number of voxels** | **Older SE** |
| --- | --- | --- | --- | --- | --- |
| **DG/CA4** | Anterior body | 146 | 7 | 139 | 9 |
|  | Posterior body | 108 | 4 | 110 | 8 |
|  | Tail | 44 | 4 | 50 | 5 |
| **CA3/2** | Anterior body | 43 | 3 | 40 | 4 |
|  | Posterior body | 27 | 1 | 30 | 4 |
|  | Tail | 17 | 1 | 16 | 1 |
| **CA1** | Anterior | 71 | 5 | 56 | 4 |
|  | Anterior body | 79 | 4 | 71 | 5 |
|  | Posterior body | 81 | 4 | 82 | 6 |
|  | Tail | 90 | 4 | 84 | 4 |
| **Subiculum** | Anterior | 57 | 4 | 49 | 3 |
|  | Anterior body | 106 | 4 | 111 | 7 |
|  | Posterior body | 95 | 3 | 102 | 5 |
|  | Tail | 39 | 4 | 38 | 3 |
| **Pre/parasubiculum** | Anterior | 28 | 3 | 25 | 3 |
|  | Anterior body | 64 | 3 | 63 | 5 |
|  | Posterior body | 55 | 2 | 59 | 5 |
|  | Tail | 20 | 1 | 20 | 2 |
| **Uncus** | Anterior | 133 | 10 | 107 | 10 |
|  | Anterior body | 130 | 18 | 113 | 13 |

SE = standard error of the mean
